# Breast arterial calcification on mammography and risk of coronary artery disease: a SCOT-HEART sub-study

**DOI:** 10.1016/j.crad.2019.01.014

**Published:** 2019-06

**Authors:** S. McLenachan, F. Camilleri, M. Smith, D.E. Newby, M.C. Williams

**Affiliations:** a Department of Radiology, Royal Infirmary of Edinburgh, Edinburgh, UK; b Edinburgh Breast Unit, Western General Hospital, Edinburgh, UK; c University of Edinburgh/British Heart Foundation Centre for Cardiovascular Science, Edinburgh, UK; d Edinburgh Imaging Facility QMRI, University of Edinburgh, Edinburgh, UK

## Abstract

**AIM:**

To assess the prevalence of breast arterial calcification (BAC) in patients who also underwent routine surveillance mammography, and to determine the association with cardiovascular risk factors, coronary artery calcification, and coronary artery disease on coronary computed tomography angiography (CCTA).

**MATERIALS AND METHODS:**

Four hundred and five female participants were identified who had undergone CCTA and subsequent mammography in the SCOT-HEART randomised controlled trial of CCTA in patients with suspected stable angina. Mammograms were assessed visually for the presence and severity of BAC.

**RESULTS:**

BAC was identified in 93 (23%) patients. Patients with BAC were slightly older (63±7 versus 59±8 years, *p<*0.001), with a higher cardiovascular risk score (19±11 versus 16±10, *p=*0.022) and were more likely to be non-smokers (73% versus 49%, *p<*0.001). In patients with BAC, coronary artery calcification was present in 58 patients (62%; relative risk [RR] 1.26, 95% confidence intervals [CI]: 1.04, 1.53; *p=*0.02), non-obstructive coronary artery disease in 58 (62%; RR 1.27, 95% CI: 1.04 to 1.54, *p=*0.02), and obstructive coronary artery disease in 19 (20%; RR 1.62, 95% CI: 0.98, 2.66; *p=*0.058). Patients without BAC were very unlikely to have severe coronary artery calcification (negative predictive value 95%) but the diagnostic accuracy of BAC to identify coronary artery disease was poor (AUC 0.547).

**CONCLUSION:**

Although BAC is associated with the presence and severity of coronary artery calcification, the diagnostic accuracy to identify patients with coronary artery disease or obstructive coronary artery disease is poor.

## Highlights

• Breast arterial calcification occurs in a fifth of patients referred for CCTA who tend to be older non-smokers.
• It is associated with higher coronary artery calcium but this is predominantly dependent on age and cardiovascular risks.
• Absence of breast arterial calcification excludes severe coronary artery calcification, negative predictive value of 95%.

## Introduction

Screening mammography is widely used to identify early breast cancer with an uptake of over 70% amongst eligible women aged between 50 and 70 years.^1^ Breast cancer is an important cause of mortality, responsible for 7% of deaths due to cancer^2^; however, worldwide the mortality from cardiovascular disease is over twice as high as that due to breast cancer. For example, in the US, there were 157,181 deaths due to ischaemic heart disease compared to 41,213 deaths due to breast cancer in 2014.^2^ The risk of cardiovascular disease is frequently underestimated for women. In addition to sex-specific risk factors for cardiovascular disease, such as menopause and pre-eclampsia, traditional risk factors including hypertension, diabetes mellitus, and smoking, are more powerful predictors for women than men.^3^ Women are also more likely to have atypical presentations of chest pain, and this has been linked to their reduced frequency of diagnosis and treatment.^3^, ^4^, ^5^, ^6^ This is a particular issue for women under the age of 55^5^ who, under current guidelines, are likely to have undergone at least one round of screening mammography.

Breast arterial calcification can be identified on screening mammography with a meta-analysis identifying a prevalence in breast cancer screening programs of 12.7%.^7^ Unlike the intimal calcification of coronary artery disease, it represents medial calcification of small mammary arteries or arterioles.^8^ In large cohorts of patients undergoing screening mammography, breast arterial calcification is associated with risk factors for cardiovascular disease,^7^ the presence of cardiovascular disease,^9^ and an increased risk of cardiovascular mortality^10^, ^11^, ^12^; however, to date, the association between breast arterial calcification and computed tomography (CT) features of coronary artery disease have only been assessed in small studies.

The Scottish COmputed Tomography of the HEART (SCOT-HEART) study is a multi-centre randomised controlled trial of the use of coronary CT angiography (CCTA) in patients with suspected coronary artery disease.^13^ It demonstrated that the use of CCTA changed the diagnosis and management of patients, which led to improved outcomes and a halving of fatal and non-fatal myocardial infarction.^13^, ^14^ In this sub-study of the SCOT-HEART trial, the prevalence of breast arterial calcification was assessed in patients who also underwent routine surveillance mammography, and the association with cardiovascular risk factors, coronary artery calcification, and coronary artery disease on CCTA was determined.

## Materials and Methods

### Study design

This is a sub-study of the SCOT-HEART trial, a multicentre randomised controlled trial of the use of CCTA in outpatients with suspected angina pectoris due to coronary artery disease (ClinicalTrials.gov, number NCT01149590).^15^ The primary results of this study have been published previously.^13^ No manuscripts on mammography in these patients have previously been published. The study was approved by the local ethics committee and informed consent obtained from all participants.

### Participants

Patients (*n=*4,146) who attended the Cardiology Outpatient Clinic were randomised to standard care or CCTA plus standard care. Of the 2,073 participants randomised to CCTA, there were 1,778 who underwent CCTA. In this sub-study, all female participants who had undergone CCTA and mammography for screening or symptomatic indications were included. Mammograms were identified on the national electronic picture archive and communications system (PACS). The ASSIGN score was used to assess cardiovascular risk. This score has been validated for use in the Scottish population and incorporates family history of cardiovascular disease, and social depravation, in addition to traditional cardiovascular risk factors.^16^

### Assessment of mammograms

Digital mammograms were reviewed blind to the results of CCTA or any other clinical factors. They were assessed by at least two of three trained radiologists using standard viewing parameters at a PACS workstation (Carestream Vue PACS, Version 11, Carestream, Rochester, NY, USA). Mediolateral oblique (MLO) and craniocaudal (CC) images were reviewed as a combined pair for each breast.

**Figure 1:**
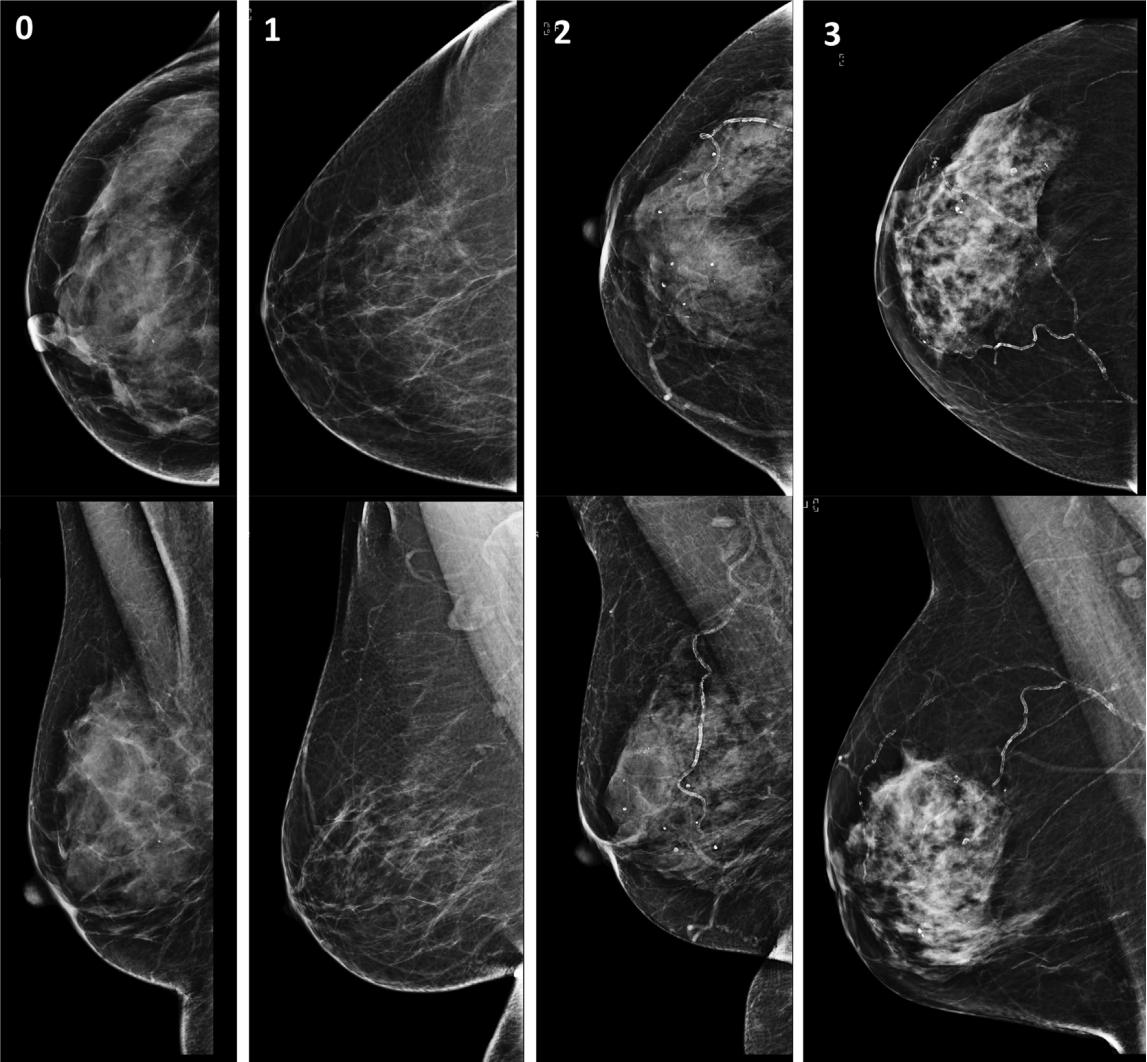
Scoring system for assessing the severity of breast arterial calcification: 0, no arterial calcification; 1, few punctate arterial calcifications with no coarse, tram track or ring calcifications; 2, coarse arterial calcification or tram track calcification in fewer than three vessels; 3, severe coarse or tram track calcification affecting three or more vessels.

The presence of any calcification on mammography was recorded and classified as vascular and non-vascular calcification. The presence and severity of breast arterial calcification was recorded. A four-point scale was used to assess the severity of breast arterial calcification (Fig 1), which was adapted from the score used by Mstafavi *et al.*^17^: 0, no vascular calcification; 1, few punctate vascular calcifications with no coarse, tram track or ring calcifications; 2, coarse vascular calcification or tram track calcification in fewer than three vessels; 3, severe coarse or tram track calcification affecting three or more vessels. Observer variability for the presence and severity scoring of breast arterial calcification was assessed in 50 separate mammograms. Per patient breast arterial calcification severity was determined by summing the breast arterial calcification score in each breast with a score of 1 considered mild, 2 moderate and ≥3 severe.

### Assessment of coronary artery calcium score and CCTA

CT was performed using 64 or 320-multidetector scanners (Brilliance 64, Philips Medical Systems, Netherlands; Biograph mCT, Siemens Germany; Aquilion ONE, Toshiba Medical Systems, Japan) at three imaging sites. Non-contrast electrocardiogram-gated CT of the heart was performed to assess coronary artery calcium score. Coronary artery calcification was assessed using the Agatston method^18^ using semi-automated software (VScore, Vital Images, Minnetonka, MN, USA or scanner console software).

Electrocardiogram-gated contrast enhanced CCTA was performed as described previously.^19^ CCTA images were assessed by two or more trained observers. The overall results of the CCTA were defined as normal (<10% luminal cross-sectional area stenosis), non-obstructive (10–70% stenosis), or obstructive coronary artery disease. Obstructive coronary artery disease was defined as a cross-sectional luminal stenosis of >70% in one or more major epicardial vessel or >50% in the left main stem. This assessment has previously been shown to have excellent intra-observer agreement and good interobserver agreement.^19^

### Clinical outcomes

Cardiovascular risk was assessed using the ASSIGN score. This score has been validated for the Scottish population and incorporates social deprivation and family history of cardiovascular disease, in addition to standard cardiovascular risk factors.^16^ Classification of clinical outcomes was performed blinded to all other results. Outcome information was obtained from the electronic Data Research and Innovation Service (eDRIS) of the National Health Service (NHS) Scotland. Where appropriate this was confirmed by review of the patient health records. The clinical endpoint for this sub-study was the occurrence of fatal or non-fatal myocardial infarction.

### Statistical analysis

Normally distributed quantitative variables are presented with mean and standard deviation. Non-normally distributed data are presented with median and interquartile range. Interobserver and intra-observer variability were assessed using kappa and weighted kappa scores. Statistical significance was assessed with Pearson's chi-squared test, Fisher's exact test and the Mann–Whitney *U*-test or Dunnett's *t*-test as appropriate. Correlation was assessed using Spearman's correlation. Hazard ratios (HR) and 95% confidence intervals (CI) are presented. Sensitivity, specificity, positive predictive value, and negative predictive value were calculated. Receiver operatory characteristic (ROC) curves were constructed to assess the area under the curve (AUC). A statistically significant difference was defined as a two-sided *p-*value <0.05.

## Results

Of the 772 female participants who underwent CCTA, 552 (72%) were eligible for screening mammography. Mammography images were available for 405 (73%) patients with a mean interval of 22±21 months between CCTA and mammography. Of these 314 (78%) were performed for screening and 91 (22%) for symptomatic assessment. There were fewer current smokers amongst the patients who had mammograms (15% versus 20%, *p=*0.002), but there were no other differences between those who did and did not undergo mammography (Table 1).

**Table 1:** Baseline characteristics and computed tomography (CT) results for participants who underwent mammography and coronary CT angiography (CCTA).

		All female participants	Female participants who had mammography	Female participants who had breast arterial calcification
Number		772	405	93
Age (years)		58±10	59±8	63±7^a^
Body mass index (kg/m^2^)		30±6	30±6	30±6
Atrial fibrillation		9 (1%)	5 (1%)	1 (1%)
Previous coronary heart disease		45 (6%)	20 (5%)	5 (5%)
Previous cerebrovascular disease		28 (4%)	13 (3%)	2 (2%)
Previous peripheral vascular disease		7 (1%)	4 (1%)	1 (1%)
Smoking status	Current	153 (20%)	62 (15%)^a^	1 (1%)^a^
	Ex-smoker	228 (30%)	122 (30%)	24 (26%)
	Non-smoker	391 (51%)	221 (55%)	68 (73%)^a^
Hypertension		254 (33%)	139 (35%)	33 (36%)
Diabetes		64 (8%)	34 (8%)	10 (11%)
Family history		364 (48%)	192 (48%)	40 (44%)
Total cholesterol		5.2±1.9	5.1±2.0	5.3±2.0
ASSIGN score		16±10.8	15.4±9.8	19.1±11.0^a^
Coronary artery calcium score		0 [0, 54]	1 [0, 58]	14 [0, 107]^a^
Any coronary artery disease on CCTA		389 (51%)	210 (52%)	58 (62%)^a^
Obstructive coronary artery disease on CCTA		105 (14%)	58 (14%)	19 (20%)

Mean±standard deviation, median and [interquartile range], *n* (%).

a *p<*0.05.

Mammographic calcification of any form was identified in 545 (68%) breasts in 318 (79%) patients. Breast arterial calcification was identified in 155 (19%) breasts in 93 (23%) patients. Four patients had unilateral mastectomy. Interobserver variability for the identification of breast arterial calcification was good (kappa of 0.799, *p<*0.001) and intra-observer variability was excellent (kappa of 0.917, *p<*0.001). For the ordinal scoring of breast arterial calcification severity, inter- and intra-observer variability were both good (kappa of 0.701, *p<*0.001 and 0.793, *p<*0.001, respectively).

Patients with breast arterial calcification on mammography were slightly older and more likely to be non-smokers compared to patients without breast arterial calcification (Table 1). Patients with breast arterial calcification also had a higher cardiovascular risk score (19±17 versus 16±10.3, *p=*0.018; Table 1). Patients with more severe breast arterial calcification were older, with a higher cardiovascular risk score, and were more likely to have a family history of coronary artery disease or be non smokers (Table 2).

**Table 2:** Baseline characteristics for participants with different levels of severity of summed breast arterial calcification score.

		Breast arterial calcification score^a^			
		None 0	Mild (1)	Moderate (2)	Severe (>=3)
Total breast arterial calcification score		0	1	2	>3
Number of patients		312 (77%)	30 (7%)	32 (8%)	31 (8%)
Age (years)		57±8	61±7^b^	63±7^b^	65±5^b^
Body mass index (kg/m^2^)		30±6	30±5	28±5	31±7
Atrial fibrillation		4 (1%)	0	1 (3%)	0
Previous coronary heart disease		15 (5%)	2 (7%)	2 (6%)	1 (3%)
Previous cerebrovascular disease		11 (4%)	1 (3%)	1 (3%)	0
Previous peripheral vascular disease		3 (1%)	0	0	1 (3%)
Smoking status	Current	61 (20%)	0	1 (3%)	0
	Ex-smoker	98 (31%)	12 (40%)	9 (28%)	3 (10%)
	Non-smoker	153 (49%)	18 (60%)	22 (69%)	28 (90%)
Hypertension		106 (34%)	5 (17%)	12 (38%)	16 (53%)^b^
Diabetes		24 (8%)	2 (7%)	3 (9%)	5 (16%)
Family history		152 (49%)	8 (29%)	16 (50%)	16 (52%)^b^
Total cholesterol		5±2.0	5.7±2.1	5.2±2.0	5.1±2.1
ASSIGN score		16±10	15±8	20±12	22±12^b^
Coronary artery calcium score		0 [0,43]	24 [0, 128]	19 [0, 97]	10 [0, 56]
Any coronary artery disease on CCTA		152 (49%)	19 (63%)	20 (63%)	19 (61%)
Obstructive coronary artery disease on CCTA		39 (12%)	9 (30%)	4 (12%)	6 (19%)

Mean±standard deviation, median and [interquartile range], number and (percentage).

CCTA, coronary computed tomography angiography.

a Summed between two breasts.

b Compared to patient with no breast arterial calcification (*p<*0.05).

Patients with breast arterial calcification were more likely to have coronary artery calcification on non-contrast CT (*n=*58, 62% versus *n=*154, 49%; relative risk [RR] 1.26; 95% CI: 1.04 to 1.53, *p=*0.018). Patients with breast arterial calcification had a higher median coronary artery calcium score (0 [interquartile range, IQR 0 to 43] versus 14 [IQR 0 to 116], *p=*0.006), but this was not independent of age or cardiovascular risk score. Patients without breast arterial calcification were very unlikely to have severe coronary artery calcification (>400 Agatston Units [AU]) with a negative predictive value of 95% (Table 2, Fig 1); however, the overall diagnostic accuracy of breast arterial calcification for identifying patients with coronary artery calcification was poor (Table 3).

**Table 3:** Diagnostic accuracy of breast arterial calcification on mammography to predict the presence of coronary artery disease on coronary computed tomography angiography (CCTA).

	TP	TN	FP	FN	Sensitivity	Specificity	PPV	NPV	AUC
Any coronary artery calcification	58	157	35	154	27	82	62	51	0.546
Coronary artery calcification (>400 AU)	8	295	85	16	33	78	9	95	0.555
Any coronary artery disease on CCTA	58	157	35	152	28	82	62	51	0.547
Obstructive coronary artery disease on CCTA	19	273	74	39	33	79	20	88	0.557

AU, Agatston units; AUC, area under the curve; FN, false negative; FP, false positive; NPV, negative predictive value; PPV, positive predictive value; TN, true negative; TP, true positive.

Patients who had breast arterial calcification had a similar frequency of aortic valve calcification (*n=*7/93, 8% versus *n=*23/312, 7%; *p=*0.718), mitral valve calcification (*n=*2/93, 2% versus *n=*6/312, 2%; *p=*0.809) and thoracic aorta calcification (*n=*19/93, 20% versus *n=*52/312, 17%; *p=*0.402) compared to those without breast arterial calcification.

**Figure 2:**
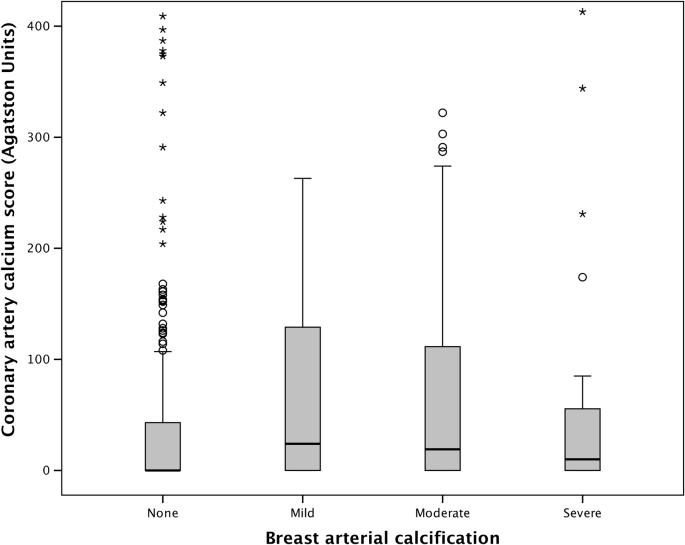
Coronary artery calcium score in patients with different severities of breast arterial calcification.

**Figure 3:**
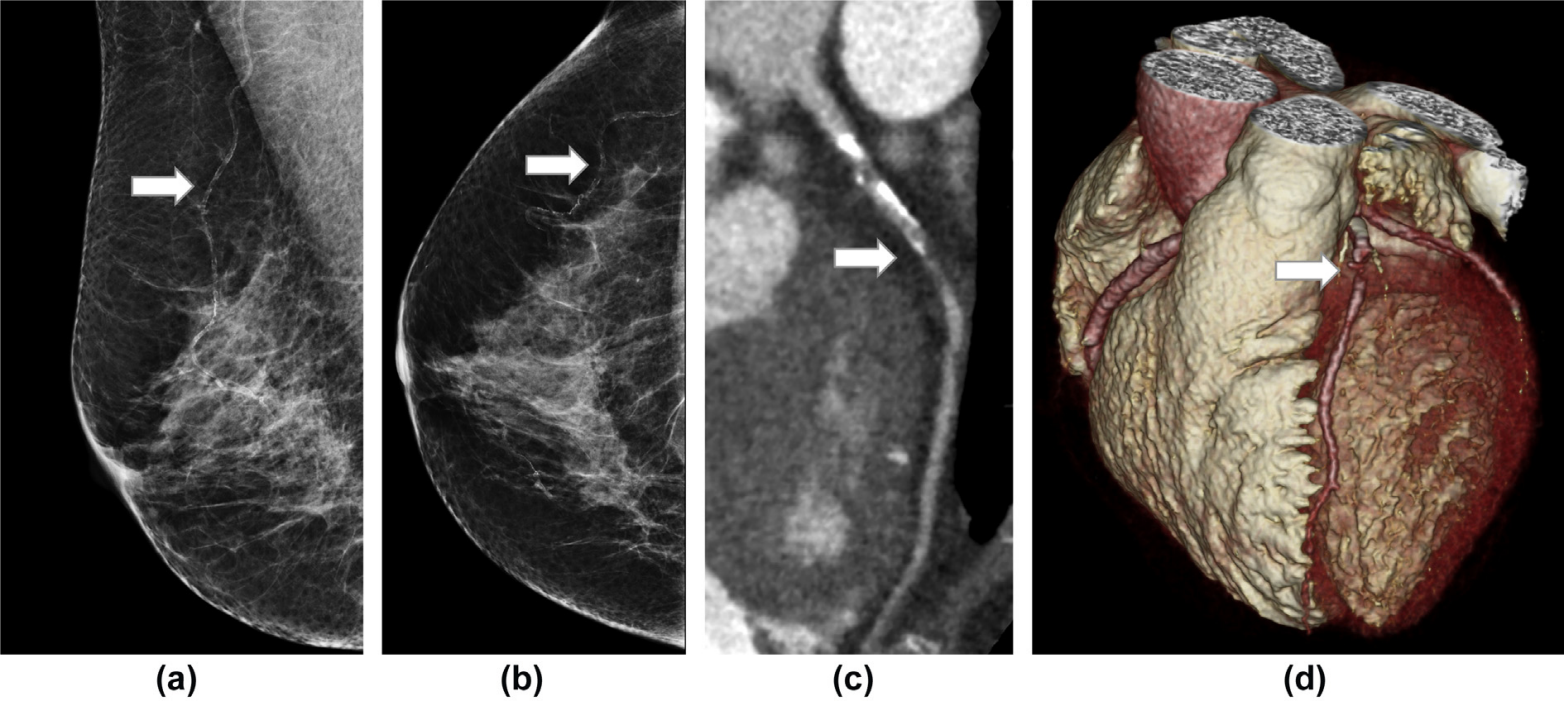
An example of a patient with severe breast arterial calcification and severe coronary artery disease. (a) Mediolateral oblique and (b) craniocaudal mammograms showing arterial calcification (arrow). (c) Curved planar reformation of the left coronary artery on CCTA showing obstructive coronary artery disease (arrow). (d) Three-dimensional CCTA reconstruction showing obstructive coronary artery disease in the left anterior descending coronary artery (arrow).

Patients with breast arterial calcification were more likely to have coronary artery disease on CCTA (Fig 3; *n=*58/93, 62% versus 152/309, 49% RR=1.27; IQR 1.04 to 1.54, *p=*0.02); however, this was not independent of age or cardiovascular risk score. Obstructive coronary artery disease on CCTA (Fig 2) appeared to be more frequent in patients with breast arterial calcification (*n=*19/93, 20% versus 39/309, 13%; RR=1.62; IQR 0.98 to 2.66; *p=*0.058). Patients with more severe breast arterial calcification were not at an increased risk of coronary artery disease or obstructive coronary artery disease (Table 2). Patients without breast arterial calcification were unlikely to have obstructive coronary artery disease with a negative predictive value of 87%, but the overall diagnostic accuracy was poor (Table 3).

The clinical outcome of fatal or non-fatal myocardial infarction at 5 years occurred in one patient who had breast arterial calcification (*n=*1/93, 1%) compared to seven patients who did not have breast arterial calcification (*n=*7/312, 2 %; *p=*0.474).

## Discussion

Breast arterial calcification occurs in one-fifth of patients referred for the assessment of suspected coronary artery disease, and the presence and severity of breast arterial calcification is associated with the cardiovascular risk score. Patients without breast arterial calcification are unlikely to have coronary artery disease on CCTA; however, the diagnostic accuracy of breast arterial calcification to identify coronary artery disease on CT is poor. Although previous studies in screening populations have shown that breast arterial calcification can identify patients at risk of coronary artery disease, mammographic breast arterial calcification is not an independent predictor of CT findings in patients with suspected angina due to coronary artery disease. Thus, the association between breast arterial calcification and cardiovascular mortality may be related to mechanisms other than the presence of coronary artery calcification or obstructive coronary artery disease.

Large cohort studies in screening populations have identified an association between breast arterial calcification and cardiovascular risk factors such as age, hypertension, hypercholesterolaemia, and diabetes mellitus.^7^, ^10^, ^11^, ^12^ Similarly, in the present study, the presence of breast arterial calcification was associated with age and cardiovascular risk score. In addition, patients with more severe breast arterial calcification had a higher cardiovascular risk score, indicating a dose-dependent response. Interestingly, the prevalence of breast arterial calcification was lower amongst smokers, both in the present study and in previous studies.^7^ This highlights an important difference in the pathophysiology of breast arterial calcification and cardiovascular disease. A systematic review of previous screening population studies identified that the prevalence of breast arterial calcification was 12.7% (95% CI: 10.4%–15.1%),^7^ whereas in the present study 23% of patients had breast arterial calcification. This likely represents the higher cardiovascular risk profile of the present population of patients with suspected coronary artery disease, compared to asymptomatic patient taking part in screening programmes.

Cohort studies of screening populations have established a link between breast arterial calcification and cardiovascular mortality, with age-adjusted hazard ratios for cardiovascular events ranging from 1.32 to 1.44.^7^, ^10^, ^11^, ^12^ In a cohort study of 12,239 women undergoing screening mammography, breast arterial calcification was associated with a 40% increase in cardiovascular mortality.^20^ Another cohort study of 12,761 women undergoing screening mammography found that breast arterial calcification was associated with coronary heart disease, ischaemic stroke, and heart failure after 25 years of follow-up.^11^ In the present study, cardiovascular outcomes were similar in patients with and without breast arterial calcification, but this is confounded by the small number of events and the relatively short duration of follow-up. The finding that breast arterial calcification is associated with cardiovascular mortality and not CT markers of coronary artery disease supports the notion of pathophysiological mechanisms of myocardial infarction in women, which are related to, but distinct from, the presence of obstructive coronary artery disease. Other mechanisms for cardiovascular mortality in women may include microvascular disease, coronary spasm, coronary artery dissection, and plaque erosion.^3^ Indeed, one-third of subsequent myocardial infarctions occur in those without obstructive coronary artery disease.^34^

Although breast arterial calcification is linked with cardiovascular risk factors and cardiovascular mortality in large screening studies, the present study does not support a link between breast arterial calcification and abnormalities in the coronary arteries on non-invasive CT imaging. The presence of breast arterial calcification has previously been assessed in small studies of patients undergoing mammography and coincidental CT for cardiac or non-cardiac indications (Electronic Supplementary Material Table S1).^17^, ^22^, ^23^, ^35^, ^36^, ^37^, ^37^, ^38^, ^39^ The largest study of coronary artery calcium score involved 499 patients and identified that the presence of breast arterial calcification on mammography was strongly associated with coronary artery calcification on subsequent CT 9 years later, with an odds ratio of 3.2 (95% CI: 1.71 to 6.04).^21^ Only two small studies have assessed the association between breast arterial calcification and the presence of coronary artery disease on CCTA.^17^, ^22^ These studies identified an association between breast arterial calcification and coronary artery disease at 10% and 50% coronary artery stenosis thresholds. Studies of other cardiac imaging techniques have shown conflicting results. Two studies have found that the severity of breast arterial calcification correlates with the severity of coronary artery disease on invasive coronary angiography,^23^, ^24^ but two other studies did not find this association.^25^, ^26^ Breast arterial calcification is also not associated with myocardial perfusion abnormalities on single photon emission CT (SPECT) imaging.^27^ Breast arterial calcification has been associated with increased carotid intima media thickness,^28^ peripheral vascular disease,^29^ reduced bone mineral density,^30^ previous or current warfarin therapy,^31^ and chronic kidney disease.^7^, ^32^ Interestingly, breast arterial calcification can regress on subsequent mammograms, highlighting that it is a dynamic process.^33^ In the present study, the absence of breast arterial calcification had a high negative predictive value, but a poor positive predictive value for the presence and severity of coronary artery calcification. The differences in results between these studies and the present study likely represent differences in demographic details between the populations.

A limitation of the present study is that not all mammography images were available due to the adoption of the digital systems at different times throughout Scotland. Mammograms from patients who moved from Scotland were not available and these patients will have been lost to follow-up. In addition, not all patients will have taken up the opportunity to undergo screening mammography. Patients who died early after CT imaging was performed, will also have been excluded from this study. Patients who underwent mammography for both screening and symptomatic purposes were included, which is likely to result in a lower age range than screening-only populations. This was a study of patients with suspected angina due to coronary artery disease rather than a cohort of patients undergoing screening mammography; therefore, no conclusions can be drawn on the utility of breast arterial calcification in identifying subclinical coronary artery disease in patients undergoing screening mammography. Further large randomised controlled trials in patients undergoing screening mammography will be required to assess the effect of the routine reporting of breast arterial calcification on subsequent cardiovascular morbidity and mortality.

In conclusion, breast arterial calcification is present in a significant proportion of patients referred for CCTA with suspected angina due to coronary artery disease; however, breast arterial calcification was not a good marker of the presence of coronary artery disease on CT in this symptomatic population. Although previous studies of screening populations have shown a link between breast arterial calcification and cardiovascular mortality, the present study did not identify a link between breast arterial calcification and CT features of coronary artery disease. This suggests that the association between breast arterial calcification and cardiovascular mortality may be driven by mechanisms other than the presence of obstructive coronary artery disease.

## Conflict of interest

MCW has performed consultancy for GE Healthcare.
