# Supplementary material for: Breast arterial calcification on mammography and risk of coronary artery disease: a SCOT-HEART sub-study
Source: Clin Radiol. 2019 Jun;74(6):421–8. doi: 10.1016/j.crad.2019.01.014 (PMC6512949; doi:10.1016/j.crad.2019.01.014)
Supplement: Multimedia component 1 [file mmc1.docx]

**Supplementary material**

*Supplementary Table 1: Previous studies of breast arterial calcification and coronary artery disease on CT*

|  | **Author** | **Year** | **Population** | **CT technique** | **N** | **Age (mean, years)** | **Diagnostic accuracy** | | | |
| --- | --- | --- | --- | --- | --- | --- | --- | --- | --- | --- |
|  |  |  |  |  |  |  | **Sensitivity** | **Specificity** | **PPV** | **NPV** |
| Coronary artery calcification | Margolies  (35) | 2016 | Referred for screening mammography and non-contrast CT for non-cardiac indications | Non-contrast non-gated CT  Ordinal calcium score > 0 | 292 | 62 | 63 | 76 | 70 | 69 |
|  | Newallo  (23) | 2015 | African American women referred for screening mammography and cardiac CT | Non-contrast, gated CT  Agatston score >100 | 204 | 53 | 67 | 86 | 40 | 95 |
|  | Pecchi  (36) | 2003 | Referred for screening mammography | Non-contrast gated CT | 74 | <65 | 70 | 86 | 95 | 40 |
|  | Maas  (22) | 2007 | Referred for screening mammography, CT at 9 years follow up | Non-contrast gated CT  Agatston score >0 | 499 | 58 | 17 | 94 | 76 | 51 |
|  | Matsumura  (37) | 2013 | Referred for screening mammography with BVC+ (98) and BVC– (104) | Non-contrast gated CT  Agatston score >400 | 202 | 59 | 54 | 12 | 99 | 56 |
|  | Chadashvili  (38) | 2016 | Referred for screening or diagnostic mammography and cardiac CT | Non-contrast gated CT  Agatston score >11 | 145 | 56 (BAC +ve) 61 (BAC –ve) | 42 | 86 | 67 | 69 |
|  | Moradi  (39) | 2014 | Referred for screening mammography and cardiac CT | Non-contrast gated CT  Agatston >0 | 150 | >40 | 57 | 80 | 23 | 95 |
| Coronary artery disease on CCTA | Newallo  (23) | 2015 | African American women referred for screening mammography and CCTA | CCTA stenosis ≥50% | 204 | 53 | 63 | 85 | 36 | 94 |
|  | Mostafavi  (17) | 2015 | Referred for screening mammography and CCTA | CCTA stenosis >10% | 100 | 66 | 34 | 97 | 83 | 78 |

*(BAC, breast arterial calcification; CACS, coronary artery calcium score; CCTA, computed tomography coronary angiography; CT computed tomography; CAD, coronary artery disease; PPV, Positive predictive value; NPV, negative predictive value*
